# Time-Course Transcriptome Analysis for Drug Repositioning in *Fusobacterium nucleatum*-Infected Human Gingival Fibroblasts

**DOI:** 10.3389/fcell.2019.00204

**Published:** 2019-09-20

**Authors:** Wenyan Kang, Zhilong Jia, Di Tang, Xiaojing Zhao, Jinlong Shi, Qian Jia, Kunlun He, Qiang Feng

**Affiliations:** ^1^Department of Human Microbiome, School and Hospital of Stomatology, Shandong University and Shandong Provincial Key Laboratory of Oral Tissue Regeneration and Shandong Engineering Laboratory for Dental Materials and Oral Tissue Regeneration, Jinan, China; ^2^Department of Periodontology, School of Stomatology, Shandong University, Jinan, China; ^3^Laboratory of Translational Medicine, Chinese PLA General Hospital, Beijing, China; ^4^Beijing Key Laboratory of Chronic Heart Failure Precision Medicine, Chinese PLA General Hospital, Beijing, China; ^5^State Key Laboratory of Microbial Technology, Shandong University, Qingdao, China

**Keywords:** *F. nucleatum*, gingival fibroblasts, RNA-seq, time-course transcriptome, drug repositioning

## Abstract

*Fusobacterium nucleatum* (*F. nucleatum*) is a crucial periodontal pathogen and human gingival fibroblasts (GFs) are the first line of defense against oral pathogens. However, the research on potential molecular mechanisms of host defense and effective treatment of *F. nucleatum* infection in GFs remains scarce. In this study, we undertook a time-series experiment and performed an RNA-seq analysis to explore gene expression profiles during the process of *F. nucleatum* infection in GFs. Differentially expressed genes (DEGs) could be divided into three coexpression clusters. Functional analysis revealed that the immune-related signaling pathways were more overrepresented at the early stage, while metabolic pathways were mainly enriched at the late stage. We computationally identified several U.S. Food and Drug Administration (FDA)-approved drugs that could protect the *F. nucleatum* infected GFs via a coexpression-based drug repositioning approach. Biologically, we confirmed that six drugs (etravirine, zalcitabine, wortmannin, calcium D-pantothenate, ellipticine, and tanespimycin) could significantly decrease *F. nucleatum*-induced reactive oxygen species (ROS) generation and block the Protein Kinase B (PKB/AKT)/mitogen-activated protein kinase signaling pathways. Our study provides more detailed molecular mechanisms of the process by which *F. nucleatum* infects GFs and illustrates the value of the cogena-based drug repositioning method and the potential therapeutic application of these tested drugs in the treatment of *F. nucleatum* infection.

## Introduction

Periodontal disease is caused by the interaction of dental plaque biofilm and the host immune system (Sanz et al., 2017). *Fusobacterium nucleatum* (*F. nucleatum*) has been confirmed as a high-frequency pathogen in periodontal disease (Moore and Moore, 1994). As a bridging bacterium, *F. nucleatum* could transfer critical periodontal pathogens, such as *Porphyromonas gingivalis* to periodontal infectious sites, recruit and activate local immune cells, and then result in tooth supporting tissue destruction (Diaz et al., 2002; Signat et al., 2011). Recent studies have confirmed that *F. nucleatum* is widely involved in the progression of colorectal cancer (Rubinstein et al., 2013), brain abscess (Kai et al., 2008), septicemia-related infections (Kroon et al., 2012), and intrauterine infections (Han et al., 2010). It has been confirmed that *F. nucleatum* could invade the tissue and play critical roles in the process of tumorigenesis and metastasis (Kostic et al., 2013; Rubinstein et al., 2013; Bullman et al., 2017; Yang et al., 2017). However, the pathogenic effect of *F. nucleatum* on oral cells has rarely been reported.

Human gingival fibroblasts (GFs) are the major constituents of gingival connective tissue, which can directly interact with pathogens and their pathogenic products and play crucial roles in regulating the host defense response (Wang et al., 2001). GFs could produce cytokines to protect the host from damage, whereas large amounts of pro-inflammatory cytokines would also cause the direct destruction of gingival tissues (Wang et al., 1999). Studies have shown that *F. nucleatum* can attack host tissues and obstruct the healing of damaged oral tissues by secreting large amounts of ammonium and butyrate (Bartold et al., 1991; Jeng et al., 1999). However, the comprehensive pathogenicity effects of *F. nucleatum* in the human oral cavity have not been determined to date.

RNA-sequencing (RNA-seq) analysis is an efficient approach to investigate the pathogenic effects of oral bacteria on oral cells. Previous RNA-seq analysis helped to elucidate host transcriptional responses to such pathogens as viruses, *Enterobacter lignolyticus*, and *Mycobacterium tuberculosis* (Kalam et al., 2017; Orellana et al., 2017; Wei et al., 2017). Ahn first used whole transcriptome analysis to explore the response of human GFs to *F. nucleatum* and found a large number of differentially expressed genes (DEGs) in aged GFs after *F. nucleatum* stimulation, which are associated with free radical scavenging, the cell cycle, and cancer (Ahn et al., 2017).

Drug repositioning, that is, exploring new indications for existing drugs that are beyond their original indications, is an increasingly attractive pattern of therapeutic discovery. Except for saving time and money, the dominant advantage of drug repurposing strategy is that existing drugs have already been confirmed in terms of their safety, dosage, and toxicity. Thus, repurposed candidate drugs can rapidly enter clinical trials compared with newly developed drugs (Ashburn and Thor, 2004). The central hypothesis of coexpression-based drug repositioning is that drugs which could reverse the gene expression signature of one specific disease can be considered as a potential drug candidate for treating the disease (Kandela and Aird, 2017). Several studies have confirmed the value of these computational approaches to identify new drug candidates for treating cancers such as cholangiocarcinoma, small-cell lung cancer, and metastatic colorectal cancer (Chen et al., 2013; Jahchan et al., 2013; Wang et al., 2013; van Noort et al., 2014; Hadley et al., 2017; Himmelstein et al., 2017; Lee et al., 2017). In this study, we applied drug repositioning to *F. nucleatum*-infected GFs.

**FIGURE 1:**
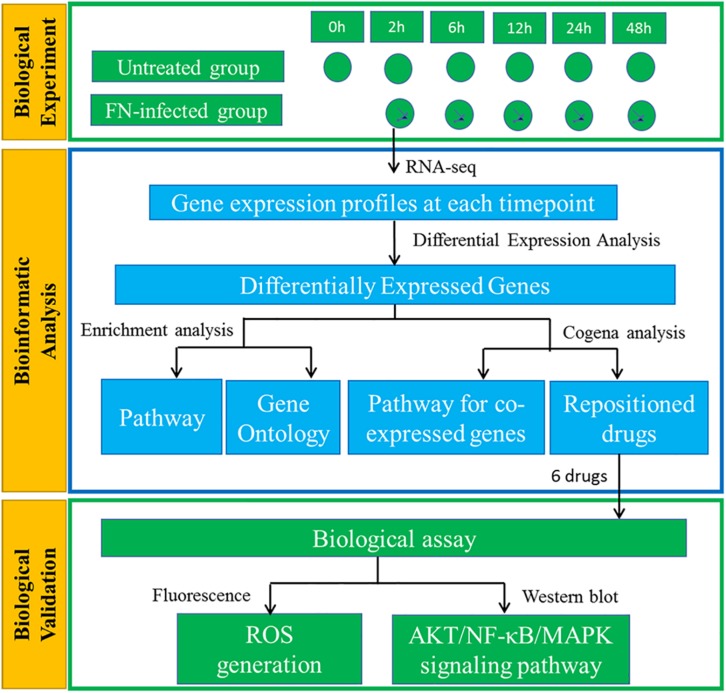
Schematic diagram of the experimental design of GFs interacting with *F. nucleatum*. GFs with or without *F. nucleatum* stimulation were subjected to RNA-seq analysis. The DEGs were identified, followed by KEGG pathway and GO enrichment analysis. Cogena analysis was used to search for the coexpression pathways and reposition the drugs to defend *F. nucleatum* infection. Finally, the potential roles of six drugs were validated with the biological experiments.

In this study, we provided a comprehensive gene expression profile of GFs along the sequence of *F. nucleatum* stimulation. Coexpressed DEGs and stage-specific enriched pathways were identified. A coexpression-based computational drug repositioning strategy was used to identify FDA-approved drugs and molecules to retard the response of GFs to *F. nucleatum* infection. We experimentally validated the effects of the drug candidates on *F. nucleatum*-induced GF intracellular reactive oxygen species (ROS) generation and Protein Kinase B (PKB/AKT), nuclear factor-κB (NF-κB), mitogen-activated protein kinase (MAPK) signaling pathway activation, which contributes to determining the fate of the cellular response to infections. The entire comprehensive experimental design is shown in Figure 1.

## Materials and Methods

### Bacterial Strains and Culture

*Fusobacterium nucleatum* strain ATCC 25586 was provided by the Shandong Provincial Key Laboratory of Oral Tissue Regeneration (Jinan, China) and was employed in this study. Stock cultures were routinely propagated in brain–heart infusion broth supplemented with 5 μg ml^–1^ hemin and 1 μg ml^–1^ menadione in an anaerobic atmosphere. The bacteria were incubated for 18 h in a constant temperature incubator at 37°C. After measuring optical density (OD) values at 600 nm, cells were harvested by centrifugation at 4500 rpm for 5 min from the fresh overnight culture, washed with phosphate-buffered solution (PBS; Solarbio, Beijing, China), and suspended in Dulbecco’s modified Eagle’s medium (DMEM; HyClone, Logan, UT, United States) for the subsequent study.

### Ethical Statements, Cell Culture, and RNA-Seq Analysis

This study was approved by the Medical Ethical Committee of the Stomatology School, Shandong University (Protocol Number: 20170101). Five subjects participating in this program were informed with this research project and signed informed consent according to the Helsinki Declaration of 1975. GFs were cultured and infected with *F. nucleatum* for 2, 6, 12, 24, and 48 h at the multiplicity of infection (MOI) equal to 100 (*F. nucleatum*:cell = 100:1), and then the total RNA of 54 samples from five individuals was extracted and subjected to the RNA-seq analysis according to our previous study (Kang et al., in press). All clean reads were mapped to the reference genome (GRCh38). The raw sequence data reported in this paper have been deposited in the Genome Sequence Archive (Wang et al., 2017) in BIG Data Center Members (2019), Beijing Institute of Genomics (BIG), Chinese Academy of Sciences, under accession number CRA001739 that are publicly accessible at http://bigd.big.ac.cn/gsa. Moreover, all raw RNA-seq data could be accessible through GEO Series accession number, GSE118691.

### Differential Expression Analysis

The Bioconductor package limma was used to detect the DEGs between control groups and *F. nucleatum*-treated groups at each time-point based on the gene expression level by the absolute of the logged fold-change of normalized expression ≥2 and an adjusted *P*-value < 0.05. Principal component analysis (PCA) was used to compare the differences among all groups. The Venn diagram visualized the overlapping genes among the DEGs of each group with the Venn package.

### Kyoto Encyclopedia of Genes and Genomes, Gene Ontology Analysis, and Computationally Drug Repositioning Analysis

Kyoto Encyclopedia of Genes and Genomes (KEGG) pathway and Gene Ontology (GO) enrichment analysis were used for the functional annotation of DEGs via the cluster Profiler Bioconductor package (Yu et al., 2012). Coexpression gene set analysis was performed using the cogena Bioconductor package and KEGG pathway from Msigdb. Cmap upregulated and downregulated gene sets were used in the cogena analysis (Jia et al., 2016). The core principle behind cogena is that coexpressed genes usually function coordinatively in the cell, and targeting these coexpressed genes will probably be more efficient for the treatment of disease. Cogena can build the connection between the coexpressed genes, pathways, and drugs, and among them, coexpressed genes were related to diseases, drugs were the candidate drugs for the disease, and pathways can uncover the drug mechanism of the action for the disease. Notably, the clustering method and the number of clusters were chosen with some instructive rules as disclosed in the original cogena paper. For example, an optimal clustering configuration should cluster a specific pathway across as few clusters as possible, ideally only one cluster. The distribution of a pathway across multiple clusters may indicate that too many clusters were selected. Unrelated pathways should be separately enriched among different clusters. In addition, the number of genes in a cluster should be at a minimum to provide an optimal maximum enrichment score (Jia et al., 2016).

### Drugs and Inhibitors

Etravirine, zalcitabine, tanespimycin, wortmannin, and calcium D-pantothenate were all purchased from Selleck (Shanghai, China) and ellipticine was purchased from MedChemExpress (MCE, Shanghai, China). All of these powders were dissolved in the appropriate solvent according to the manufacturer’s instructions.

### Cell Viability Assay

Cultured GFs were seeded in a 96-well plate (8,000 cells/well) and incubated overnight at 37°C in a CO_2_ incubator. After cell adhesion and extension, drugs at various concentrations were diluted in DMEM and pretreated cells for 24 h. The effects of etravirine, zalcitabine, wortmannin, calcium D-pantothenate, ellipticine, and tanespimycin on GFs’ viability were determined by cell-counting kit-8 according to the manufacturer’s instructions (CCK-8, Dojindo, Kumamoto, Japan). Briefly, 100 μl of medium containing 10 μl of test reagents was added to each well, and the plates were incubated for 2.5 h at 37°C in a CO_2_ incubator. The OD value was measured at a wavelength of 450 nm using a microplate reader (SPECTROstar Nano, BMG Labtech, Offenburg, Germany). The experiments were performed in sextuplicate and repeated three times in GFs from three different donors.

### Measurement of Intracellular ROS

Intracellular ROS levels were detected with a 2′,7′-dichlorofluorescein diacetate (DCFH-DA) assay according to the cell reactive oxygen detection kit (BestBio, Shanghai, China) following the manufacturer’s instructions. GFs were seeded in 96-well plates and pretreated with drugs for 24 h and then stimulated with *F. nucleatum* (MOI = 100) for another 24 h. Cells were incubated with DCFH-DA (1:1000) for 20 min at 37°C in a cell incubator with darkness. A fluorescence microplate reader was used to determine intracellular ROS production. The fluorescence intensity value was measured at 488/535 nm wavelength using a fluorescence microplate reader (VICTOR X2, PerkinElmer, Waltham, MA, United States). The experiments were performed in sextuplicate and repeated three times in GFs from three different donors.

### Western Blot Analysis

Gingival fibroblasts were seeded in 6-well plates and pretreated with drugs for 24 h and then stimulated with *F. nucleatum* (MOI = 100) for 30 min. Cells were harvested with RIPA lysis buffer containing 1% protease inhibitor and 1% phosphatase inhibitor (Solarbio, Beijing, China). Protein concentrations were measured according to bicinchoninic acid (BCA) protein assays and 10 μg/lane proteins were loaded onto 10% sodium dodecyl sulfate-polyacrylamide gel electrophoresis (SDS-PAGE) gels and transferred to polyvinylidene fluoride (PVDF) membranes (Millipore, Billerica, MA, United States). The membranes were incubated with primary antibodies at 4°C overnight at a dilution of 1:1000–10,000 according to the manufacturer’s instructions (Supplementary Table S1), and then incubated with horseradish peroxidase-conjugated secondary antibodies (1:10,000; Proteintech, Chicago, IN, United States) for 1 h at room temperature. Western blotting images were captured using a FluorChem E System (Amersham Imager 600; GE Healthcare Life Sciences, Pittsburgh, PA, United States). ImageJ 1.44 software (NIH, Bethesda, MD, United States) was used to quantify the protein expression level. The experiments were repeated three times in GFs from three different donors.

### Quantitative Real-Time Polymerase Chain Reaction

Gingival fibroblasts were seeded in 96-well plates and pretreated with drugs for 24 h and then stimulated with *F. nucleatum* (MOI = 100) for another 24 h. Total RNA was extracted with TRIzol^®^ (CWBIO, Beijing, China) and the mRNA was reverse-transcribed to cDNA according to the instruction of the HiFiScript cDNA Synthesis kit (CWBIO, Beijing, China). Quantitative real-time polymerase chain reaction (qRT-PCR) was performed according to the manufacturer’s manual of UltraSYBR Mixture (CWBIO, Beijing, China) by a LightCycler 96 Real-Time PCR System (Roche, Basel, Switzerland). Data were analyzed by the 2^(–ΔΔ*Ct)*^ method. The sequences of the primers used are shown in Supplementary Table S2. The experiments were repeated three times in GFs from three different donors.

### Statistical Analysis

All biological data for validating the candidate drugs are presented as the mean ± standard deviation (SD). Tests were analyzed using GraphPad Prism software (version 6, MacKiev Software, Boston, MA, United States), and differences among more than two groups were analyzed by one-way analysis of variance (ANOVA) followed by Tukey’s honestly significant difference (HSD) comparison test. A *P*-value < 0.05 considered to be statistically significant. The R code and data to reproduce all the figures and tables are available via the link https://github.com/zhilongjia/Fn_HGFcell.

## Results

### RNA-Seq Analysis of *F. nucleatum*-Stimulated GFs at Each Time Point

**FIGURE 2:**
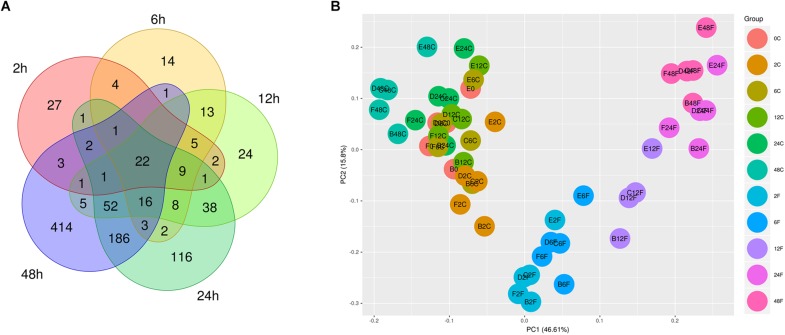
Venn diagram of all the DEGs at each time-point. **(A)** GFs were treated with *F. nucleatum* (MOI = 100) at 2, 6, 12, 24, and 48 h, and 22 core overlapping DEGs were identified among the five time points. **(B)** PCA of 54 samples based on the expression of all DEGs. The samples are from five donors. The first letter (B, C, D, E, or F) in the sample name represents the donor ID, the number in the middle represents the time point, and the last letter C or F represents control and *F. nucleatum*, respectively. In the legend, C and F represent the control and *F. nucleatum*, respectively.

The differential expression analysis for each two groups was carried out at the first step between the *F. nucleatum*-treated group and the control group at each time point. Seventy-nine, 98, 197, 458, and 707 DEGs were identified at 2, 6, 12, 24, and 48 h, respectively, as visualized by a Venn diagram (Figure 2A). Among all the DEGs at each time point, 22 core genes, such as IL6, SOD2, and PTGS2, were significantly upregulated in the whole process of *F. nucleatum* infection (Supplementary Table S3). The expression profiling of 971 genes, the union of all DEGs at each time point, was visualized with a heatmap (Supplementary Figure S1). As shown in Supplementary Figure S1, there were three highly separated sample clusters in the hierarchy structure of clusters; the first cluster was all the control groups at each time-point and 0 h, and the second cluster was the treated groups from 2, 6, and 12 h, separated from the third cluster, the treated groups from 24 and 48 h. The distribution of all 54 samples in the two-dimensional space projected by PCA of the expression profile of the 971 DEGs verified this observation with a view toward group separation (Figure 2B). The separation of different groups was clear. The control and *F. nucleatum*-infected groups appeared separately along the first axis, and the samples in the control groups were located nearer to each other without differences in time series, while samples within each infected group of 2, 6, 12, 24, and 48 h aggregated, respectively, and separated from one another.

### Pathway and GO Analysis of DEGs at Each Time Point

**FIGURE 3:**
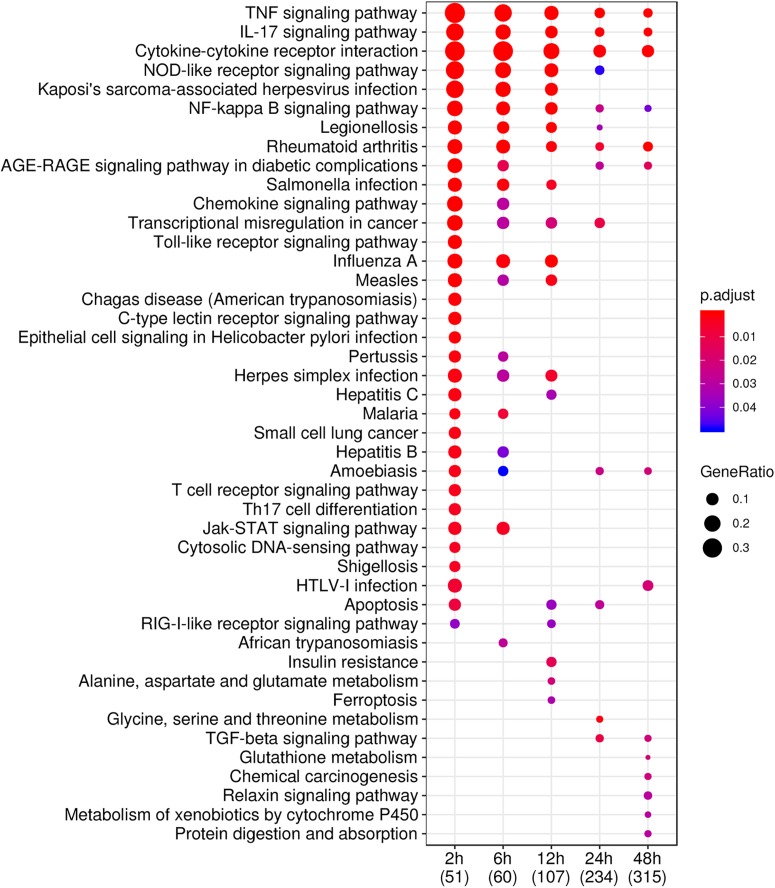
KEGG pathway enrichment analysis of DEGs at each time-point. GFs were stimulated by *F. nucleatum* at 2, 6, 12, 24, and 48 h, and 51, 60, 107, 234, and 315 DEGs (entrez ID) were identified, and the top 39 pathways are shown. The size of the dot represents the degree of enrichment and the color indicates the degree of statistical significance.

The functional analysis of DEGs at each time-point on pathway and GO level presented pathways and GO changes over time. The results of KEGG pathway analysis are shown in Figure 3. Specifically, the Tumour necrosis factor (TNF)-signaling pathway, IL-17 signaling pathway, cytokine–cytokine receptor interaction, NF-κB signaling pathway, and rheumatoid arthritis pathway were continuously activated and the magnitude of enrichment slowly decreased with time, although the number of DEGs increased with time. Interestingly, several relevant pathways acted at different stages. For example, the bacteria (such as legionellosis, pertussis, *Helicobacter pylori*) and virus (such as salmonella, influenza, measles, herpes simplex, hepatitis B/C, human T Lymphotropic virus (HTLV)-I) infection pathways, chemokine signaling pathway, Toll-like receptor (TLR) signaling pathway, apoptosis signaling pathway, and jak-STAT signaling pathway were activated at the early stage (before 12 h), while the transforming growth factor (TGF)-beta signaling pathway, glutathione metabolism, protein digestion, and absorption signaling pathway were activated at the late stage (after 12 h). These results indicated that drastic changes occurred in GFs from 12 to 24 h after infection with *F. nucleatum*. The GO overrepresentation analysis is illustrated in Supplementary Figure S2. Cytokine and growth factor activities were highly enriched at all times points, but the extent of enrichment decreased over time. Transmembrane transporter, wnt signal, and extracellular matrix structural constituent ontologies were overrepresented at the late stage. The analysis of GO also confirmed that a variety of genes functioned approximately 12 h after the infection of *F. nucleatum*.

### Time Course Coexpression Pathway Analysis

**FIGURE 4:**
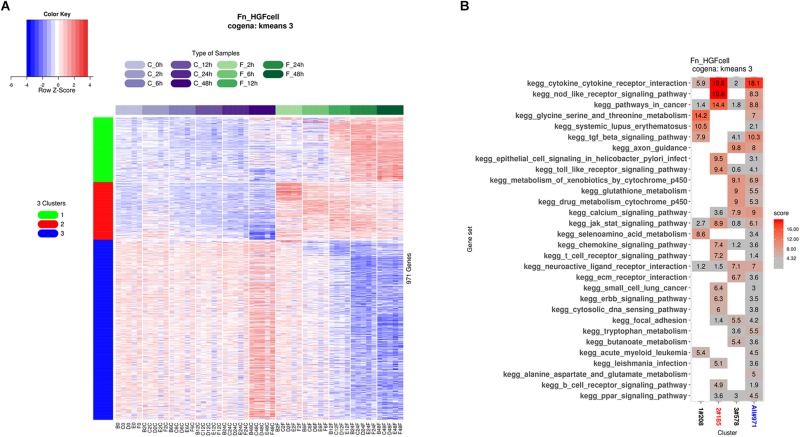
Time course coexpression pathway analysis. **(A)** GFs were stimulated by *F. nucleatum* at 2, 6, 12, 24, and 48 h, and all united DEGs were shown in the heatmap and all these DEGs were clearly divided into three clusters. The DEGs in cluster 1 were upregulated after *F. nucleatum* stimulation at 12 h. The DEGs in cluster 2 were upregulated immediately after *F. nucleatum* stimulation. The DEGs in cluster 3 were downregulated after *F. nucleatum* stimulation at 12 h. **(B)** The DEGs of three clusters were subjected to coexpression KEGG pathway analysis, and the degrees of enrichment of each pathway are presented with the enrichment scores.

Biologically, coexpressed genes tend to have similar functions at the cytological level. We identified coexpressed genes in the DEGs and performed pathway analysis of these coexpressed genes using cogena, a tool developed in our previous study (Jia et al., 2016). We found that the DEGs could be divided into three clusters (Figure 4A). Specifically, considering the regulation direction of the DEGs, genes in cluster 1 were upregulated after 12 h of *F. nucleatum* stimulation, and genes in cluster 2 were upregulated immediately, while genes in cluster 3 were down-regulated after 12 h (Figure 4A). KEGG pathway analysis revealed that genes in cluster 2 were highly enriched in the cytokine–cytokine receptor signaling pathway, NOD-like receptor signaling pathway, and TLR signaling pathway, while genes in clusters 1 and 3 were mainly enriched in metabolism-related pathways. Notably, TGF-β signaling and systemic lupus erythematosus pathways were overrepresented in cluster 1. The immune response in GFs to *F. nucleatum* was triggered in <2 h of *F. nucleatum* infection, while the normal metabolism process in GFs was severely disturbed after 12 h (Figure 4B). The results suggested that the major response was altered over time during *F. nucleatum* infection in GFs.

### Computational Drug Repositioning for Treating *F. nucleatum*-Infected GFs

**FIGURE 5:**
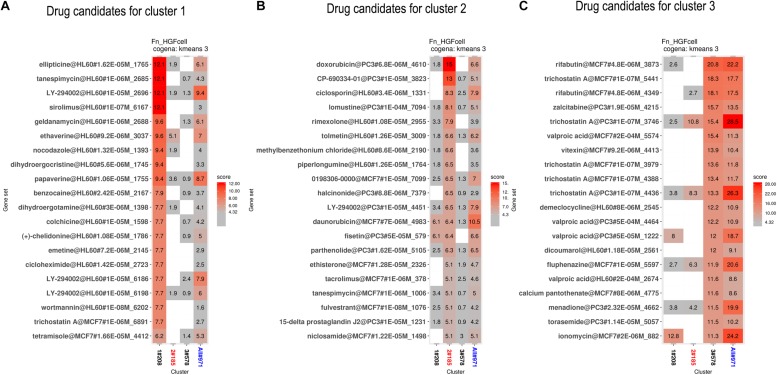
Computational drug repositioning for the coexpressed genes. The enrichment scores of candidate drugs. **(A)** Drug candidates, such as ellipticine, tanespimycin, etravirine, and wortmannin, mainly target DEGs in cluster 1. **(B)** Drug candidates (such as tanespimycin) mainly target DEGs in cluster 2. **(C)** Drug candidates (such as zalcitabine and pantothenic acid) mainly target DEGs in cluster 3.

We performed drug repositioning analysis to identify potential drug candidates by a cogena-based computational drug repositioning pipeline. The core of cogena is to identify drugs that can largely reverse the regulation direction of a cluster of coexpressed DEGs in GFs affected by the infection of *F. nucleatum*, and the results of computational drug repositioning for each cluster are presented in Figure 5. Not unexpectedly, several antibiotics and immunosuppressants were rediscovered, and some drugs that are not antibiotics or immunosuppressants were enriched in the results of cogena drug repositioning analysis.

Drug candidates mainly targeting coexpressed genes in cluster 1 are shown in Figure 5A. Among these drugs, we found some were relevant to anti-bacterial activity or immunity. Sirolimus, ranked 4th, is an immunosuppressant, especially in preventing the rejection of kidney transplants. Geldanamycin, ranked 5th, is a benzoquinone ansamycin antitumor antibiotic with moderate anti-microbial, anti-fungal, and anti-protozoa activity (DeBoer et al., 1970). To the best of our knowledge, tanespimycin, a derivative of geldanamycin, is being studied in the treatment of cancer but not in antibiotics. Etravirine is a drug used for the treatment of human immunodeficiency virus (HIV). Wortmannin and LY-294002 are PI3K inhibitors. Ellipticine, ranked first, is an inhibitor of topoisomerase II. Trichostatin A is an antifungal antibiotic.

Drug candidates for cluster 2 are listed in Figure 5B. Ciclosporin, tolmetin, and tacrolimus, which are immunosuppressants, were ranked 3rd, 6th, and 16th, respectively. Rimexolone, ranked 5th, is a glucocorticoid steroid used to treat inflammation in the eye. Methylbenzethonium chloride, ranked 7th, possesses antiseptic and anti-infective properties. Tanespimycin, ranked 17th, appeared again.

Drug candidates for cluster 3 are listed in Figure 5C. Rifabutin, the first drug, is an antibiotic used to treat tuberculosis and prevent and treat the mycobacterium avium complex. Zalcitabine is approved for the treatment of HIV. Demeclocycline can treat various types of bacterial infections. Trichostatin A appeared in all three clusters. Pantothenic acid is also known as vitamin B5.

From this drug repositioning approach, we predicted a list of candidate drugs with the efficacy against *F. nucleatum*. This list covered a wide variety of drugs, including antineoplastic drugs (e.g., ellipticine), immunity inhibitors (sirolimus), Hsp90 inhibitors (tanespimycin and geldanamycin), PI3K inhibitors (wortmannin, LY-294002), and HIV drugs (zalcitabine and etravirine), suggesting that these agents may prevent or treat the diseased effect of *F. nucleatum* infection by altering the gene expression signature in the infected GFs.

### Biological Validation of the Candidate Drugs *in vitro*

Biologically experimental validation of several drugs’ roles in against *F. nucleatum* infection and protecting the cells, including the effect of candidate drugs on GF proliferation, and *F. nucleatum*-elevated intracellular ROS generation and NF-κB, MAPK, and AKT signaling pathway activation were performed. Among the predicted drugs, six drugs, etravirine, zalcitabine, wortmannin, calcium D-pantothenate, ellipticine, and tanespimycin, were selected to be tested due to the scale of biological experiments.

**FIGURE 6:**
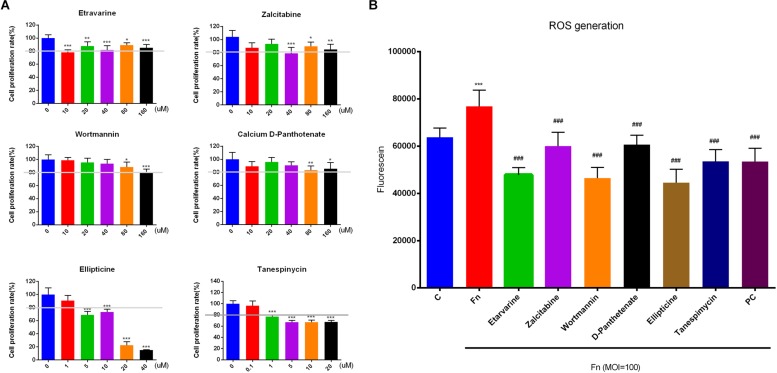
Effects of candidate drugs on GF proliferation and *F. nucleatum*-induced ROS generation. **(A)** GFs were treated with etravirine, zalcitabine, wortmannin, and calcium D-pantothenate at 10, 20, 40, 80, and 160 μM, and ellipticine (1, 5, 10, 20, and 40 μM) and tanespimycin (0.1, 1, 5, 10, and 20 μM) were used to treat cells for 24 h. The proliferation rates of GFs were detected by CCK-8. The white line represents 80% inhibitory concentration. **(B)** Etravirine (80 μM), zalcitabine (80 μM), wortmannin (80 μM), calcium D-pantothenate (80 μM), ellipticine (10 μM), and tanespimycin (10 μM) were pretreated GFs for 24 h before *F. nucleatum* infection (MOI = 100) for another 24 h. All drugs could significantly decrease *F. nucleatum*-elevated intracellular ROS generation in GFs. The histogram represents the mean ± *SD* (*n* = 6). C, control; Fn, *F. nucleatum*-treated group; PC, positive control. Statistical analyses were performed by one-way ANOVA with Tukey’s multiple-comparison test. ^∗^*P* < 0.05; ^∗∗^*P* < 0.01; ^∗∗∗^*P* < 0.001 compared with the control group.^ ###^*P* < 0.001 compared with the *F. nucleatum*-treated group.

We confirmed that all six drugs dose-dependently inhibited primary GF proliferation. GFs were more tolerant to etravirine, zalcitabine, wortmannin, and calcium D-pantothenate and 10 μM etravirine, 40 μM zalcitabine, 80 μM wortmannin, and 80 μM calcium D-pantothenate significantly inhibited GF proliferation. On the other hand, ellipticine and tanespimycin were sensitive to GF proliferation, and 1 μM tanespimycin and 0.1 μM ellipticine presented inhibitory effects on cell growth. To ensure a better effect of the candidate drug on *F. nucleatum*-stimulated GFs, we selected the drug concentration that could maintain the survival of GFs over 80% compared with the negative control group for the subsequent experiments. Thus, etravirine (80 μM), zalcitabine (80 μM), wortmannin (80 μM), calcium D-pantothenate (80 μM), ellipticine (10 μM), and tanespimycin (10 μM) were used to pretreat GFs before *F. nucleatum* stimulation (Figure 6A).

*Fusobacterium nucleatum* at the MOI of 100 significantly elevated the GF intracellular ROS level at 24 h stimulation, and etravirine, zalcitabine, wortmannin, calcium D-pantothenate, ellipticine, and tanespimycin, significantly reduced the *F. nucleatum*-elevated intracellular ROS level (Figure 6B). As ROS play key roles in regulating cell proliferation, apoptosis, and the inflammatory response (Rasul et al., 2013; Leavy, 2014), our results indicated that these drugs had the potential to develop as an adjuvant drug in treating the disease induced by abundant ROS generation.

**FIGURE 7:**
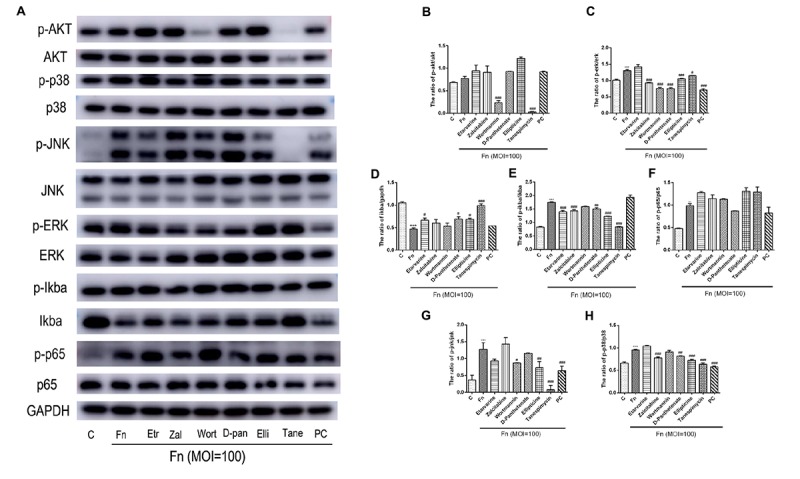
Effects of candidate drugs on *F. nucleatum*-activated NF-κB, MAPK, and AKT signaling pathways in GFs. **(A)** The protein levels of AKT, phosphorylated AKT, p38, phosphorylated p38, JNK, phosphorylated JNK, ERK, phosphorylated ERK, IκBα, phosphorylated IκBα, NF-κB p65, and phosphorylated NF-κB p65 were detected by western blotting. The relative expression of **(B)** phosphorylated AKT/AKT, **(C)** phosphorylated ERK/ERK, **(D)** IκBα/GAPDH, **(E)** phosphorylated IκBα/IκBα, **(F)** phosphorylated NF-κB p65/NF-κBp65, **(G)** phosphorylated JNK/JNK, and **(H)** phosphorylated p38/p38 was quantified by ImageJ. Histograms represent means ± *SD* (*n* = 3). C, control; Fn, *F. nucleatum*-treated group; PC, positive control. Statistical analyses were performed using one-way ANOVA with Tukey’s multiple-comparison test. ^∗∗^*P* < 0.01 and ^∗∗∗^*P* < 0.001 compared with the control group. ^#^*P* < 0.05, ^##^*P* < 0.01, and ^###^*P* < 0.001 compared with the *F. nucleatum*-treated group.

Furthermore, we used western blot assay to detect the roles of candidate drugs in the *F. nucleatum*-activated AKT, NF-κB, and MAPK signaling pathways. The results showed that *F. nucleatum* at the MOI of 100 could significantly activate the NF-κB, MAPK, and AKT signaling pathways at 1 h of stimulation (Figure 7). *F. nucleatum* activated pathways related to cell proliferation by elevating the abundance ratio of p-AKT/AKT and p-ERK/ERK (Figure 7A). All drugs, except etravirine, could effectively block the ERK signaling pathway. Wortmannin and tanespimycin could significantly decrease the *F. nucleatum*-activated Akt signaling pathway, while other drugs could not (Figures 7A–C). *F. nucleatum* activated the NF-κB signaling pathway by increasing the proportion of p-p65/p65, p-IκBα/IκBα, and degrading IκBα (Figure 7A). Etravirine, calcium D-pantothenate, ellipticine, and especially tanespimycin could inhibit *F. nucleatum*-induced IκBα degradation (Figure 7D). All drugs except wortmannin could significantly dephosphorylate *F. nucleatum*-induced IκBα, and notably, the ratio of p-IκBα/IκBα in the tanespimycin-treated group was equivalent to that in the control group (Figure 7E). Zalcitabine could significantly decrease the phosphorylation level of IκBα while partly increasing the IκBα protein level. For the protein level of p-p65, calcium D-pantothenate showed a slightly inhibitory effect of *F. nucleatum*-elevated p-p65 levels with no statistical significance. The results indicated that the candidate drugs mainly regulated the NF-κB signaling pathway by increasing IκBα protein levels rather than decreasing phosphorylated p65 levels (Figures 7A,D–F). In addition, *F. nucleatum* significantly activated the MAPK signaling pathway by increasing the proportion of p-jnk/jnk, p-ERK/ERK, and p-p38/p38 protein (Figure 7A). Zalcitabine, wortmannin, calcium D-pantothenate, ellipticine, and tanespimycin could significantly block the ERK and p38 signaling pathway by reducing the phosphorylated protein levels (Figures 7A,C,H). Etravirine, wortmannin, ellipticine, and tanespimycin could largely reverse the *F. nucleatum*-activated JNK signaling pathway by decreasing the p-JNK level, while zalcitabine and calcium D-pantothenate could not (Figures 7A,G). The results demonstrated that the tested drugs played relevant roles in defending against *F. nucleatum* infection and protecting GFs via *F. nucleatum*-activated AKT, NF-κB, and MAPK signaling pathways.

## Discussion

Our study provides a novel, quantitative, accurate, and comprehensive time course gene expression landscape in human GFs following *F. nucleatum* infection through efficient utilization of RNA-seq technology, and this research further confirms that *F. nucleatum* regulates GFs’ biological properties by influencing the immune response in the early stage while interfering with cell metabolism in the late stage. Moreover, we use a computational drug repositioning approach to seek drugs to defend *F. nucleatum* and examine the effect of the selected drugs in protecting GFs after *F. nucleatum* stimulation. Previous studies have confirmed that *F. nucleatum* could significantly trigger ROS generation in GFs at 2 h postinfection, which is critical in regulating the host defense response (Ahn et al., 2016). Due to the universality of periodontal disease and the high invasive ability of *F. nucleatum*, the pathogenic mechanism of *F. nucleatum* to GFs is an important proposition in the etiology of periodontal disease. However, the previous transcriptome analysis on *F. nucleatum* to GFs was a cross-sectional study on a single time point of *F. nucleatum*-treated and untreated oral cells, which is difficult to determine the cytological changes during the invasion of pathogenic bacteria because the pathogenic process is a dynamic process of persistent interaction between the host and pathogens. In the present study, we used an RNA-seq strategy to assess the time-course impact of *F. nucleatum* on the cellular fate of GFs and clearly distinguished the stage-specific DEGs and signaling pathways. We also identified specific targeted drugs to these pathways and validated the role of these drugs in alleviating *F. nucleatum*-induced ROS generation and AKT, NF-κB, and MAPK signaling pathway activation.

RNA sequencing has been widely applied in many differential gene expression studies (Oshlack et al., 2010; Ozsolak and Milos, 2011). In recent years, RNA-seq, as a comprehensive and systematic approach to defining the transcriptome of an organism with minimal bias (Humphrys et al., 2013), has attracted considerable attention from scholars for its widespread use in various cell types and experimental settings without specific probes or cross-hybridization issues (Mortazavi et al., 2008; Bruno et al., 2010). RNA-seq has been used to predict and prioritize candidate cancer neoantigens and dual RNA-seq has been developed to unravel the complex interplay between bacterial virulence and host response (Karasaki et al., 2017; Marsh et al., 2019). However, current RNA-seq profiling of eukaryotic gene expression in bacterially infected cells is generally limited to a single time point. Indeed, cell growth is a constant dynamic process, even after bacterial infection, which suggests that time-course transcriptome analysis is needed in the bacterial infected cells.

Our experimental design is intended to characterize the temporal dynamics of gene expression in GFs during the *F. nucleatum* stimulation at 2, 6, 12, 24, and 48 h to illuminate the series of progressive biological mechanisms involved in *F. nucleatum* infection. By comparing GF gene expression between *F. nucleatum*-infected GFs and control groups at each time point, we identified 79, 98, 197, 458, and 707 DEGs at 2, 6, 12, 24, and 48 h, resulting in 971 united DEGs after *F. nucleatum* stimulation. In addition, we identified that the early-stage DEGs were enriched in immune-related signaling pathways, such as the MAPK signaling pathway, chemokine signaling pathway, and T-cell receptor signaling pathway, while large numbers of late stage DEGs were enriched in metabolite-related pathways, such as glycerophospholipid metabolism, *o*-glycan biosynthesis, and the insulin signaling pathway. These data suggested that during *F. nucleatum* infection, the host immune system was activated to defend the foreigner invader at first, while excessive pathogenic bacteria could evade the host immune response and perturb the host normal metabolic process.

Bacterial infection could trigger the production of ROS and then regulate intracellular signaling and host defense (Torres et al., 2006). ROS, mainly produced from the mitochondrial electron transport of aerobic respiration, are reactive molecules and free radicals derived from molecular oxygen (Rovin et al., 1990) and play roles in promoting cell apoptosis and the inflammatory response (Rasul et al., 2013; Leavy, 2014). *F. nucleatum* has a greater capacity to metabolize oxygen molecules (Diaz et al., 2002) and could activate NOX1 and NOX2 in GFs, which leads to an oxygen-limited environment that facilitates attachment of the strict anaerobe *P. gingivalis* to GFs and consequently increases GF apoptosis (Ahn et al., 2016). Our previous study confirmed that *F. nucleatum* could increase the production of ROS and sequentially induce apoptosis and inflammatory cytokine production in GFs (Kang et al., in press). In this study, we validated the effects of our predicated candidate drugs on ROS generation after *F. nucleatum* stimulation and the results indicated that all of the drugs are effective in inhibiting *F. nucleatum*-elevated ROS production, which suggested that these drugs have the potential to protect *F. nucleatum*-infected GFs.

Gingival fibroblasts, as the first physical line of defense against oral microflora, could locally orchestrate immune reactions by recognizing the specific pathogen-associated molecular patterns through their respective TLRs (Handfield et al., 2008). *F. nucleatum* is considered to be an opportunistic pathogen that could participate in the disease process when environmental conditions allow it. From our RNA-seq data, it is notable that among the 13,658 genes tested, IL-8, IL-6, CCL2, SOD2, and PTGS2 expression was significantly increased at all stages in *F. nucleatum* infection. IL-8 is a key chemokine for the accumulation of neutrophils and has been confirmed to play key roles in the acute inflammatory response (Eftang et al., 2012). IL-6 could alter osteocyte signaling toward osteoblasts and elicit periodontitis through facilitating osteoclastogenesis and bone resorption (Kwan et al., 2004; Bakker et al., 2014). GFs could secrete IL-6 and CCL2 via activating TLR4 signaling, MAPK, and NF-κB pathways, resulting in the progression of periodontitis (Nishikawa et al., 2017). PTGS2, namely, cyclooxygenase 2 (COX-2), could be induced and act on arachidonic acid to form prostaglandins (PGs), which are pivotal in the pathogenesis of periodontitis (Noguchi et al., 1999; Morton and Dongari-Bagtzoglou, 2001). SOD2, a main antioxidant enzyme, could maintain ROS homeostasis under inflammatory conditions and is upregulated in periodontitis (Yoon et al., 2018). In the current study, we confirmed that the six selected drugs play different roles in regulating *F. nucleatum*-elevated genes expression: etarvirine significantly down regulated the expression of IL-6, CCL2, and PTGS2; zalcitabine synergistically enhanced IL6 and IL-8 expression; wortmannin promoted IL-8 and CCL2 expression, while decreased the expression of SOD2 and PTGS2; D-pantothenate showed no effective roles except for elevating the expression of PTGS2; ellipticine played synergistically roles on elevating the expression of IL-6 and SOD2, while down regulated the expression of CCL2; and tanespimycin significantly decreased the expression of IL-6, SOD2, and PTGS2 (Supplementary Figure S3). Based on the above results, we speculate that single drug is difficult to reverse inflammatory response induced by *F. nucleatum*, which suggests that drug combination therapy may be more effective to treat *F. nucleatum* infection diseases.

It has been reported that AKT, MAPK, and NF-κB signaling pathways could be activated in inflammatory conditions in various cell types (Thompson and Van Eldik, 2009; Oita et al., 2010; Mendonca et al., 2018). Our RNA-seq data and a previous study confirmed that *F. nucleatum* could significantly activate the AKT, NF-κB, and MAPK (ERK, JNK, and p38) signaling pathways. In this study, our biological experiments confirmed that these drugs could largely block the *F. nucleatum*-activated ERK, JNK, and p38 MAPK signaling pathways by decreasing the phosphorylation protein levels of p-ERK, p-JNK, and p-MAPK. Regarding the NF-κB signaling pathway, we found that almost all of the drugs, except wortmannin, could decrease pathway activation by increasing the degradation of IκBα, rather than decreasing the phosphorylation level of p65. Wortmannin and tanespimycin could weaken AKT signaling pathway activation. As a result, based on our computational prediction and experimental validation *in vitro*, tanespimycin can be considered as a candidate drug to treat *F. nucleatum-*infected GFs with a higher probability than other tested drugs. However, our study is limited due to lack of the detailed *in vitro* and *in vivo* biological validation experiments to evaluate the effects of the candidate drugs on GF biological properties variation. In the future, we will comprehensively validate the biological effects of each drug on *F. nucleatum*-infected GFs through various biological experiments, such as cell proliferation, apoptosis, migration, differentiation assays, and further test the roles of the drugs on *F. nucleatum-*infected rat models.

## Conclusion

In summary, our study concludes that *F. nucleatum* could time-dependently regulate the gene expression of human GFs and affect GFs’ biological properties by disturbing the immune-related signaling pathways in the early stage and interfering with cell metabolism in the late stage. The potential candidate drugs (etravirine, zalcitabine, wortmannin, calcium D-pantothenate, ellipticine, and tanespimycin) identified via a cogena-based computational drug repositioning approach could significantly decrease *F. nucleatum*-induced ROS generation and inhibit AKT, MAPK, and NF-κB signaling pathways to protect the *F. nucleatum*-infected GFs. Our study provides a new method for the treatment of *F. nucleatum*-induced GF infection.

## Data Availability Statement

The datasets generated for this study can be found in the Genome Sequence Archive in BIG Data Center, CRA001739; GEO database, GSE118691.

## Ethics Statement

The studies involving human participants were reviewed and approved by the Medical Ethical Committee of the Stomatology School, Shandong University (Protocol Number: 20170101). The patients/participants provided their written informed consent to participate in this study.

## Author Contributions

QF, XZ, and KH designed and supervised the study. WK and DT collected the samples and performed the experiments. WK, ZJ, JS, and QJ analyzed and interpreted the data. WK, ZJ, and QF wrote the manuscript. All authors commented on this manuscript.

## Conflict of Interest

All authors declared six patent applications related to etravirine, zalcitabine, tanespimycin, wortmannin, calcium D-pantothenate, and ellipticine that were submitted to the Chinese Patent Office.

## Supplementary Material

FIGURE S1 Heatmap of all the united 971 DEGs in 54 samples. The first letter in the sample name represents the donor ID, the middle number represents the time, and the last letters C and F represent control and *F. nucleatum*, respectively.

FIGURE S2 GO analysis of DEGs at each time-point. GFs were stimulated by *F. nucleatum* at 2, 6, 12, 24, and 48 h, and 77, 93, 182, 416, and 621 DEGs (entrez ID) were identified compared with each control group. The size of the dot represents the degree of enrichment, and the color indicates a significant difference.

FIGURE S3 The gene expression of IL-6, IL-8, CCL2, SOD2, and PTGS2. Etravirine (80 μM), zalcitabine (80 μM), wortmannin (80 μM), calcium D-pantothenate (80 μM), ellipticine (10 μM), and tanespimycin (10 μM) were pretreated GFs for 24 h before *F. nucleatum* infection (MOI = 100) for another 24 h. The genes expression of IL-6 **(A)**, IL-8 **(B)**, CCL2 **(C)**, SOD2 **(D)**, and PTGS2 **(E)** was detected by RT-PCR. The histogram represents the mean ± *SD* (*n* = 3). C, control; Fn, *F. nucleatum*-treated group; PC, positive control. Statistical analyses were performed by one-way ANOVA with Tukey’s multiple-comparison test. ^∗∗∗^*P* < 0.001 compared with the control group.^ #^*P* < 0.05, ^##^*P* < 0.01, and ^###^*P* < 0.001 compared with the *F. nucleatum*-treated group.

TABLE S1 Primary antibodies for western blot.

TABLE S2 Primers sequences for quantitative real-time PCR (qRT-PCR).

TABLE S3 Twenty-two core DEGs of time-series *F. nucleantum*-infected GFs.

## References

[B1] Ahn S. H. Chun S. Park C. Lee J. H. Lee S. W. Lee T. H. (2017). Transcriptome profiling analysis of senescent gingival fibroblasts in response to *Fusobacterium nucleatum* infection. *PLoS One* 12:e0188755. 10.1371/journal.pone.0188755 29190775 PMC5708803

[B2] Ahn S. H. Song J. E. Kim S. Cho S. H. Lim Y. K. Kook J. K. (2016). NOX1/2 activation in human gingival fibroblasts by *Fusobacterium nucleatum* facilitates attachment of Porphyromonas gingivalis. *Arch. Microbiol.* 198 573–583. 10.1007/s00203-016-1223-7 27071620

[B3] Ashburn T. T. Thor K. B. (2004). Drug repositioning: identifying and developing new uses for existing drugs. *Nat. Rev. Drug Discov.* 3 673–683. 10.1038/nrd1468 15286734

[B4] Bakker A. D. Kulkarni R. N. Klein-Nulend J. Lems W. F. (2014). IL-6 alters osteocyte signaling toward osteoblasts but not osteoclasts. *J. Dent. Res.* 93 394–399. 10.1177/0022034514522485 24492932

[B5] Bartold P. M. Gully N. J. Zilm P. S. Rogers A. H. (1991). Identification of components in *Fusobacterium nucleatum* chemostat-culture supernatants that are potent inhibitors of human gingival fibroblast proliferation. *J. Periodontal Res.* 26 314–322. 10.1111/j.1600-0765.1991.tb02069.x 1831499

[B6] BIG Data Center Members (2019). Database resources of the BIG data center in 2019. *Nucleic Acids Res.* 47 D8–D14. 10.1093/nar/gky993 30365034 PMC6323991

[B7] Bruno V. M. Wang Z. Marjani S. L. Euskirchen G. M. Martin J. Sherlock G. (2010). Comprehensive annotation of the transcriptome of the human fungal pathogen Candida albicans using RNA-seq. *Genome Res.* 20 1451–1458. 10.1101/gr.109553.110 20810668 PMC2945194

[B8] Bullman S. Pedamallu C. S. Sicinska E. Clancy T. E. Zhang X. Cai D. (2017). Analysis of *Fusobacterium* persistence and antibiotic response in colorectal cancer. *Science* 358 1443–1448. 10.1126/science.aal5240 29170280 PMC5823247

[B9] Chen M. H. Lin K. J. Yang W. L. Kao Y. W. Chen T. W. Chao S. C. (2013). Gene expression-based chemical genomics identifies heat-shock protein 90 inhibitors as potential therapeutic drugs in cholangiocarcinoma. *Cancer Am. Cancer Soc.* 119 293–303. 10.1002/cncr.27743 22810956

[B10] DeBoer C. Meulman P. A. Wnuk R. J. Peterson D. H. (1970). Geldanamycin, a new antibiotic. *J. Antibiot.* 23 442–447. 10.7164/antibiotics.23.442 5459626

[B11] Diaz P. I. Zilm P. S. Rogers A. H. (2002). *Fusobacterium nucleatum* supports the growth of *Porphyromonas gingivalis* in oxygenated and carbon-dioxide-depleted environments. *Microbiology* 148 467–472. 10.1099/00221287-148-2-467 11832510

[B12] Eftang L. L. Esbensen Y. Tannaes T. M. Bukholm I. R. Bukholm G. (2012). Interleukin-8 is the single most up-regulated gene in whole genome profiling of *H. pylori* exposed gastric epithelial cells. *BMC Microbiol.* 12:9. 10.1186/1471-2180-12-9 22248188 PMC3292955

[B13] Hadley D. Pan J. El-Sayed O. Aljabban J. Aljabban I. Azad T. D. (2017). Precision annotation of digital samples in NCBI’s gene expression omnibus. *Sci. Data* 4:170125. 10.1038/sdata.2017.125 28925997 PMC5604135

[B14] Han Y. W. Fardini Y. Chen C. Iacampo K. G. Peraino V. A. Shamonki J. M. (2010). Term stillbirth caused by oral *Fusobacterium nucleatum*. *Obstet. Gynecol.* 115 442–445. 10.1097/AOG.0b013e3181cb9955 20093874 PMC3004155

[B15] Handfield M. Baker H. V. Lamont R. J. (2008). Beyond good and evil in the oral cavity: insights into host-microbe relationships derived from transcriptional profiling of gingival cells. *J. Dent. Res.* 87 203–223. 10.1177/154405910808700302 18296603 PMC2633067

[B16] Himmelstein D. S. Lizee A. Hessler C. Brueggeman L. Chen S. L. Hadley D. (2017). Systematic integration of biomedical knowledge prioritizes drugs for repurposing. *eLife* 6:e26726. 10.7554/eLife.26726 28936969 PMC5640425

[B17] Humphrys M. S. Creasy T. Sun Y. Shetty A. C. Chibucos M. C. Drabek E. F. (2013). Simultaneous transcriptional profiling of bacteria and their host cells. *PLoS One* 8:e80597. 10.1371/journal.pone.0080597 24324615 PMC3851178

[B18] Jahchan N. S. Dudley J. T. Mazur P. K. Flores N. Yang D. Palmerton A. (2013). A drug repositioning approach identifies tricyclic antidepressants as inhibitors of small cell lung cancer and other neuroendocrine tumors. *Cancer Discov.* 3 1364–1377. 10.1158/2159-8290.CD-13-0183 24078773 PMC3864571

[B19] Jeng J. H. Chan C. P. Ho Y. S. Lan W. H. Hsieh C. C. Chang M. C. (1999). Effects of butyrate and propionate on the adhesion, growth, cell cycle kinetics, and protein synthesis of cultured human gingival fibroblasts. *J. Periodontol.* 70 1435–1442. 10.1902/jop.1999.70.12.1435 10632518

[B20] Jia Z. Liu Y. Guan N. Bo X. Luo Z. Barnes M. R. (2016). Cogena, a novel tool for co-expressed gene-set enrichment analysis, applied to drug repositioning and drug mode of action discovery. *BMC Genomics* 17:414. 10.1186/s12864-016-2737-8 27234029 PMC4884357

[B21] Kai A. Cooke F. Antoun N. Siddharthan C. Sule O. (2008). A rare presentation of ventriculitis and brain abscess caused by *Fusobacterium nucleatum*. *J. Med. Microbiol.* 57 668–671. 10.1099/jmm.0.47710-0 18436604

[B22] Kalam H. Fontana M. F. Kumar D. (2017). Alternate splicing of transcripts shape macrophage response to Mycobacterium tuberculosis infection. *PLoS Pathog.* 13:e1006236. 10.1371/journal.ppat.1006236 28257432 PMC5352146

[B23] Kandela I. Aird F. (2017). Replication study: discovery and preclinical validation of drug indications using compendia of public gene expression data. *eLife* 6:e17044. 10.7554/eLife.17044 28100397 PMC5245962

[B24] Kang W. Jia Z. Tang D. Zhang Z. Gao H. He K. (2019). *Fusobacterium nucleatum* facilitates apoptosis, ROS generation, and inflammatory cytokine production by activating AKT/MAPK and NF-κB signaling pathways in human gingival fibroblasts. *Oxid Med Cell Longev* (in press). 10.1155/2019/1681972 PMC6815639 31737164

[B25] Karasaki T. Nagayama K. Kuwano H. Nitadori J. I. Sato M. Anraku M. (2017). Prediction and prioritization of neoantigens: integration of RNA sequencing data with whole-exome sequencing. *Cancer Sci.* 108 170–177. 10.1111/cas.13131 27960040 PMC5329159

[B26] Kostic A. D. Chun E. Robertson L. Glickman J. N. Gallini C. A. Michaud M. (2013). *Fusobacterium nucleatum* potentiates intestinal tumorigenesis and modulates the tumor-immune microenvironment. *Cell Host Microbe* 14 207–215. 10.1016/j.chom.2013.07.007 23954159 PMC3772512

[B27] Kroon E. Arents N. A. Halbertsma F. J. (2012). Septic arthritis and osteomyelitis in a 10-year-old boy, caused by *Fusobacterium nucleatum*, diagnosed with PCR/16S ribosomal bacterial DNA amplification. *BMJ Case Rep.* 2012:bcr1220115335. 10.1136/bcr.12.2011.5335 22605875 PMC3369400

[B28] Kwan T. S. Padrines M. Theoleyre S. Heymann D. Fortun Y. (2004). IL-6, RANKL, TNF-alpha/IL-1: interrelations in bone resorption pathophysiology. *Cytokine Growth. Factor. Rev* 15 49–60. 10.1016/j.cytogfr.2003.10.005 14746813

[B29] Leavy O. (2014). Inflammation: regulating ROS. *Nat. Rev. Immunol.* 14:357. 10.1038/nri3685 24798369

[B30] Lee B. K. Tiong K. H. Chang J. K. Liew C. S. Abdul R. Z. Tan A. C. (2017). DeSigN: connecting gene expression with therapeutics for drug repurposing and development. *BMC Genomics* 18:934. 10.1186/s12864-016-3260-7 28198666 PMC5310278

[B31] Marsh J. W. Hayward R. J. Shetty A. Mahurkar A. Humphrys M. S. Myers G. (2019). Dual RNA-seq of chlamydia and host cells. *Methods Mol. Biol.* 2042 123–135. 10.1007/978-1-4939-9694-0_9 31385273

[B32] Mendonca P. Taka E. Bauer D. Reams R. R. Soliman K. (2018). The attenuating effects of 1,2,3,4,6 penta-O-galloyl-beta-d-glucose on pro-inflammatory responses of LPS/IFNgamma-activated BV-2 microglial cells through NFkB and MAPK signaling pathways. *J. Neuroimmunol.* 324 43–53. 10.1016/j.jneuroim.2018.09.004 30236786 PMC6245951

[B33] Moore W. E. Moore L. V. (1994). The bacteria of periodontal diseases. *Periodontol 2000* 5 66–77. 10.1111/j.1600-0757.1994.tb00019.x 9673163

[B34] Mortazavi A. Williams B. A. McCue K. Schaeffer L. Wold B. (2008). Mapping and quantifying mammalian transcriptomes by RNA-Seq. *Nat. Methods.* 5 621–628. 10.1038/nmeth.1226 18516045 PMC13303166

[B35] Morton R. S. Dongari-Bagtzoglou A. I. (2001). Cyclooxygenase-2 is upregulated in inflamed gingival tissues. *J. Periodontol.* 72 461–469. 10.1902/jop.2001.72.4.461 11338298

[B36] Nishikawa Y. Kajiura Y. Lew J. H. Kido J. I. Nagata T. Naruishi K. (2017). Calprotectin induces IL-6 and MCP-1 production via toll-like receptor 4 signaling in human gingival fibroblasts. *J. Cell. Physiol.* 232 1862–1871. 10.1002/jcp.25724 27925202

[B37] Noguchi K. Shitashige M. Ishikawa I. (1999). Involvement of cyclooxygenase-2 in interleukin-1alpha-induced prostaglandin production by human periodontal ligament cells. *J. Periodontol.* 70 902–908. 10.1902/jop.1999.70.8.902 10476899

[B38] Oita R. C. Ferdinando D. Wilson S. Bunce C. Mazzatti D. J. (2010). Visfatin induces oxidative stress in differentiated C2C12 myotubes in an Akt- and MAPK-independent, NFkB-dependent manner. *Pflugers. Arch.* 459 619–630. 10.1007/s00424-009-0752-1 19898975

[B39] Orellana R. Chaput G. Markillie L. M. Mitchell H. Gaffrey M. Orr G. (2017). Multi-time series RNA-seq analysis of *Enterobacter* lignolyticus SCF1 during growth in lignin-amended medium. *PLoS One* 12:e0186440. 10.1371/journal.pone.0186440 29049419 PMC5648182

[B40] Oshlack A. Robinson M. D. Young M. D. (2010). From RNA-seq reads to differential expression results. *Genome Biol.* 11:220. 10.1186/gb-2010-11-12-220 21176179 PMC3046478

[B41] Ozsolak F. Milos P. M. (2011). RNA sequencing: advances, challenges and opportunities. *Nat. Rev. Genet.* 12 87–98. 10.1038/nrg2934 21191423 PMC3031867

[B42] Rasul A. Bao R. Malhi M. Zhao B. Tsuji I. Li J. (2013). Induction of apoptosis by costunolide in bladder cancer cells is mediated through ROS generation and mitochondrial dysfunction. *Molecules.* 18 1418–1433. 10.3390/molecules18021418 23348995 PMC6269911

[B43] Rovin B. H. Wurst E. Kohan D. E. (1990). Production of reactive oxygen species by tubular epithelial cells in culture. *Kidney Int.* 37 1509–1514. 10.1038/ki.1990.142 2163466

[B44] Rubinstein M. R. Wang X. Liu W. Hao Y. Cai G. Han Y. W. (2013). *Fusobacterium nucleatum* promotes colorectal carcinogenesis by modulating E-cadherin/beta-catenin signaling via its FadA adhesin. *Cell Host Microbe.* 14 195–206. 10.1016/j.chom.2013.07.012 23954158 PMC3770529

[B45] Sanz M. Beighton D. Curtis M. A. Cury J. A. Dige I. Dommisch H. (2017). Role of microbial biofilms in the maintenance of oral health and in the development of dental caries and periodontal diseases. Consensus report of group 1 of the Joint EFP/ORCA workshop on the boundaries between caries and periodontal disease. *J. Clin. Periodontol.* 44(Suppl. 18), S5–S11. 10.1111/jcpe.12682 28266109

[B46] Signat B. Roques C. Poulet P. Duffaut D. (2011). *Fusobacterium nucleatum* in periodontal health and disease. *Curr. Issues Mol. Biol.* 13 25–36. 21220789

[B47] Thompson W. L. Van Eldik L. J. (2009). Inflammatory cytokines stimulate the chemokines CCL2/MCP-1 and CCL7/MCP-3 through NFkB and MAPK dependent pathways in rat astrocytes [corrected]. *Brain Res.* 1287 47–57. 10.1016/j.brainres.2009.06.081 19577550 PMC2725204

[B48] Torres M. A. Jones J. D. Dangl J. L. (2006). Reactive oxygen species signaling in response to pathogens. *Plant Physiol.* 141 373–378. 10.1104/pp.106.079467 16760490 PMC1475467

[B49] van Noort V. Scholch S. Iskar M. Zeller G. Ostertag K. Schweitzer C. (2014). Novel drug candidates for the treatment of metastatic colorectal cancer through global inverse gene-expression profiling. *Cancer Res.* 74 5690–5699. 10.1158/0008-5472.CAN-13-3540 25038229

[B50] Wang P. L. Oido-Mori M. Fujii T. Kowashi Y. Kikuchi M. Suetsugu Y. (2001). Heterogeneous expression of Toll-like receptor 4 and downregulation of Toll-like receptor 4 expression on human gingival fibroblasts by *Porphyromonas gingivalis* lipopolysaccharide. *Biochem. Biophys. Res. Commun.* 288 863–867. 10.1006/bbrc.2001.5842 11688988

[B51] Wang P. L. Shirasu S. Shinohar M. Azuma Y. Daito M. Yasuda H. (1999). IL-10 inhibits Porphyromonas gingivalis LPS-stimulated human gingival fibroblasts production of IL-6. *Biochem. Biophys. Res. Commun.* 263 372–377. 10.1006/bbrc.1999.1381 10491300

[B52] Wang Y. Song F. Zhu J. Zhang S. Yang Y. Chen T. (2017). GSA: genome sequence archive. *Genomics Proteomics Bioinformatics* 15 14–18. 10.1016/j.gpb.2017.01.001 28387199 PMC5339404

[B53] Wang Y. C. Deng N. Chen S. Wang Y. (2013). Computational study of drugs by integrating omics data with Kernel methods. *Mol. Inform.* 32 930–941. 10.1002/minf.201300090 27481139

[B54] Wei J. Zhang H. Li X. Li Q. Ma Z. Bai J. (2017). Transcriptional profiling of host cell responses to encephalomyocarditis virus (EMCV). *Virol. J.* 14:45. 10.1186/s12985-017-0718-4 28259172 PMC5336634

[B55] Yang Y. Weng W. Peng J. Hong L. Yang L. Toiyama Y. (2017). *Fusobacterium nucleatum* increases proliferation of colorectal cancer cells and tumor development in mice by activating Toll-like receptor 4 signaling to nuclear factor-kappaB, and up-regulating expression of MicroRNA-21. *Gastroenterology* 152 851–866.e24. 10.1053/j.gastro.2016.11.018 27876571 PMC5555435

[B56] Yoon Y. Kim T. J. Lee J. M. Kim D. Y. (2018). SOD2 is upregulated in periodontitis to reduce further inflammation progression. *Oral Dis.* 24 1572–1580. 10.1111/odi.12933 29972711

[B57] Yu G. Wang L. G. Han Y. He Q. Y. (2012). clusterProfiler: an R package for comparing biological themes among gene clusters. *OMICS* 16 284–287. 10.1089/omi.2011.0118 22455463 PMC3339379

