# Supplementary figures and images for: Time-Course Transcriptome Analysis for Drug Repositioning in Fusobacterium nucleatum-Infected Human Gingival Fibroblasts
Source: Front Cell Dev Biol. 2019 Sep 20;7:204. doi: 10.3389/fcell.2019.00204 (PMC6771468; doi:10.3389/fcell.2019.00204)

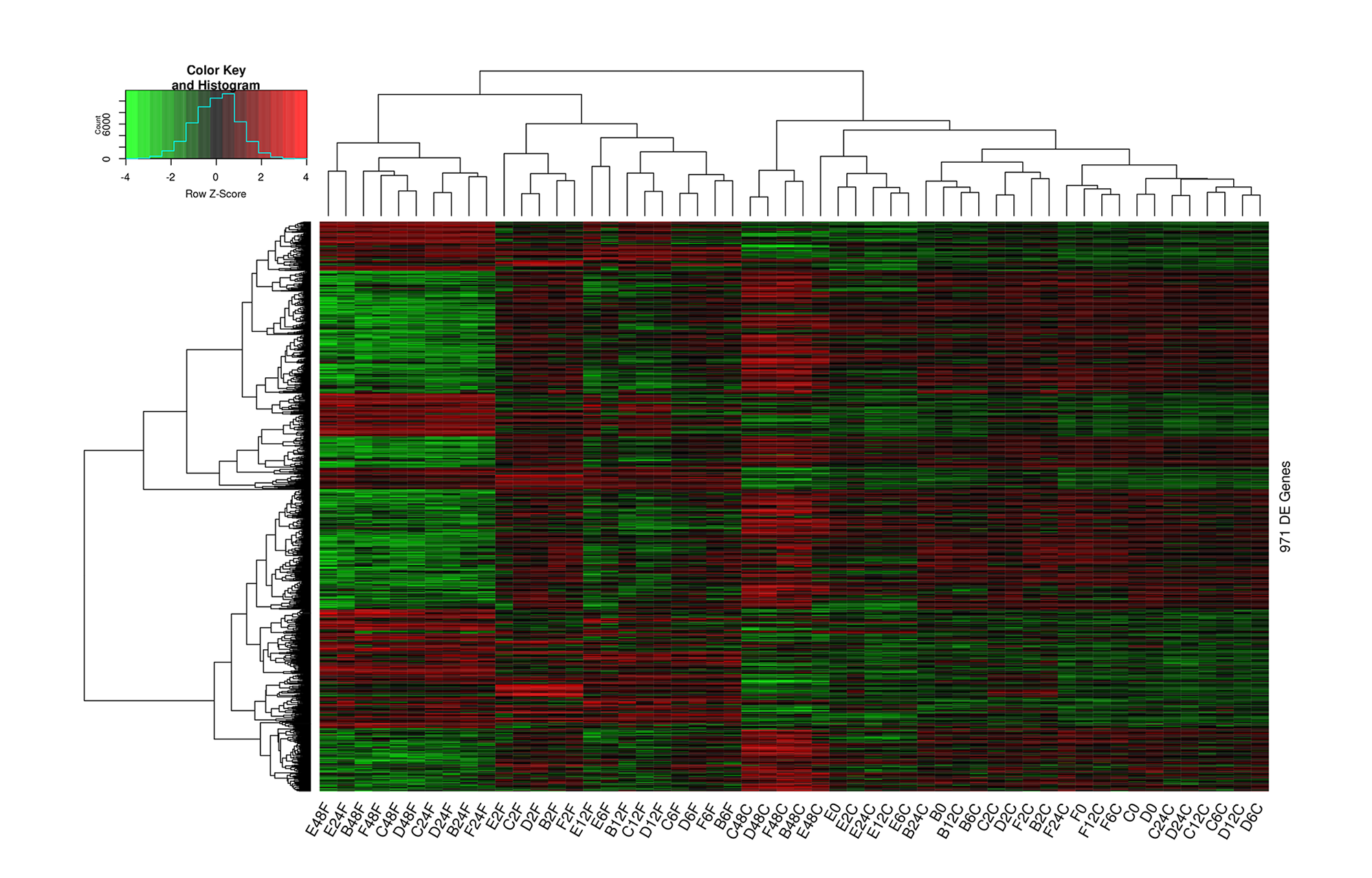

Supplement: FIGURE S1 — Heatmap of all the united 971 DEGs in 54 samples. The first letter in the sample name represents the donor ID, the middle number represents the time, and the last letters C and F represent control and F. nucleatum, respectively. [file Image_1.TIF]

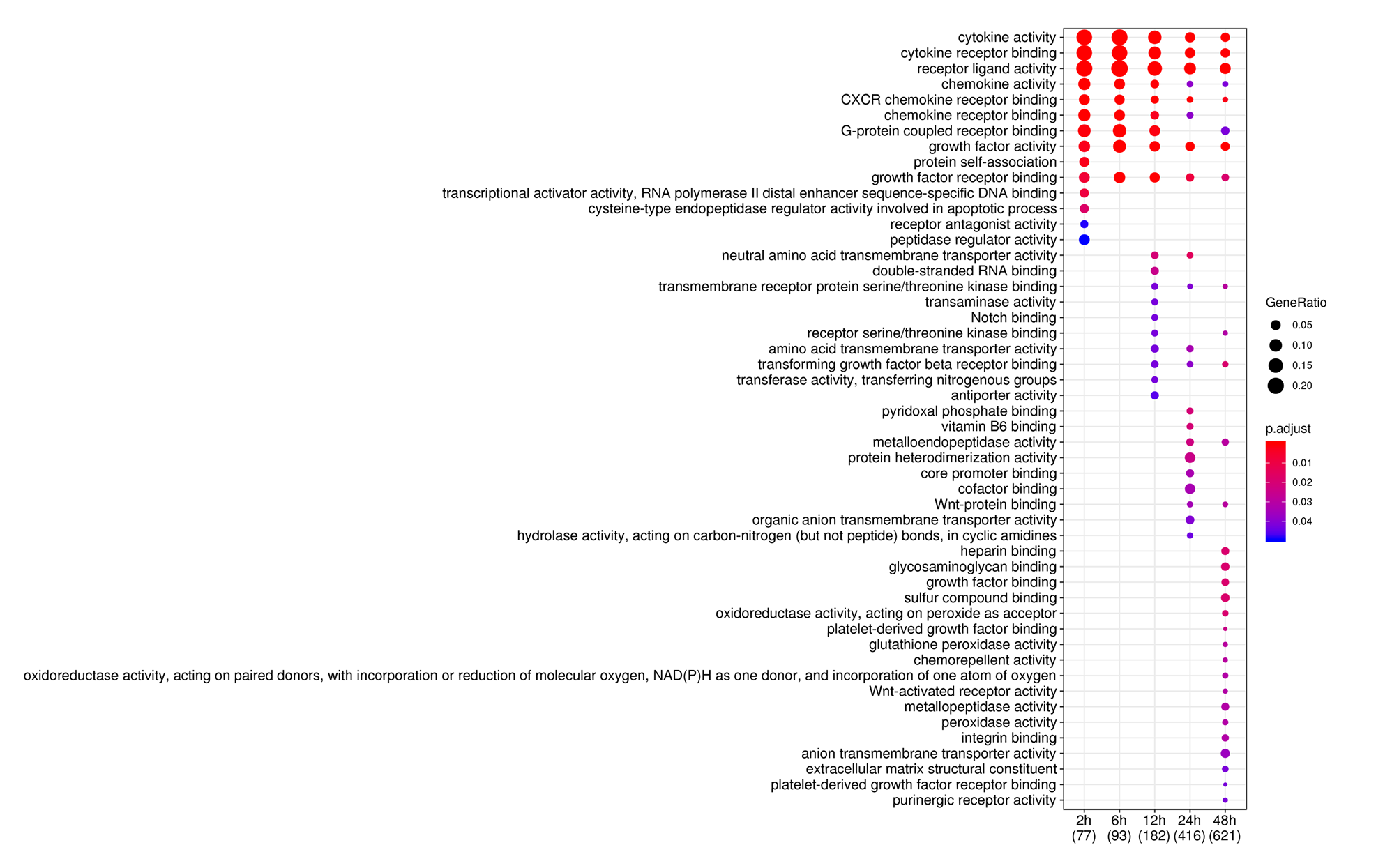

Supplement: FIGURE S2 — GO analysis of DEGs at each time-point. GFs were stimulated by F. nucleatum at 2, 6, 12, 24, and 48 h, and 77, 93, 182, 416, and 621 DEGs (entrez ID) were identified compared with each control group. The size of the dot represents the degree of enrichment, and the color indicates a significant difference. [file Image_2.TIF]

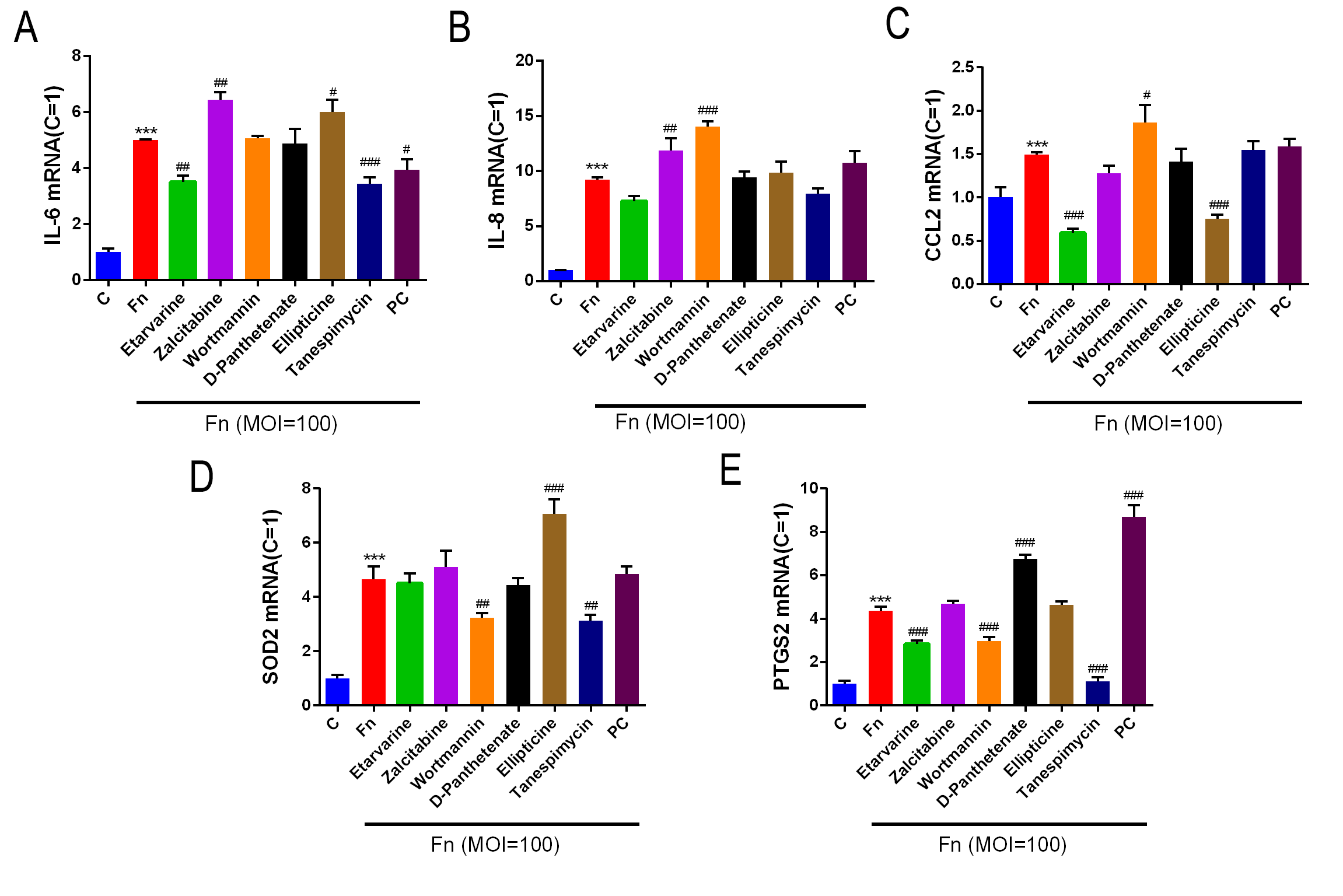

Supplement: FIGURE S3 — The gene expression of IL-6, IL-8, CCL2, SOD2, and PTGS2. Etravirine (80 μM), zalcitabine (80 μM), wortmannin (80 μM), calcium D-pantothenate (80 μM), ellipticine (10 μM), and tanespimycin (10 μM) were pretreated GFs for 24 h before F. nucleatum infection (MOI = 100) for another 24 h. The genes expression of IL-6 (A), IL-8 (B), CCL2 (C), SOD2 (D), and PTGS2 (E) was detected by RT-PCR. The histogram represents the mean ± SD (n = 3). C, control; Fn, F. nucleatum-treated group; PC, positive control. Statistical analyses were performed by one-way ANOVA with Tukey’s multiple-comparison test. ∗∗∗P < 0.001 compared with the control group. #P < 0.05, ##P < 0.01, and ###P < 0.001 compared with the F. nucleatum-treated group. [file Image_3.TIF]
