# Supplementary material for: Time-Course Transcriptome Analysis for Drug Repositioning in Fusobacterium nucleatum-Infected Human Gingival Fibroblasts
Source: Front Cell Dev Biol. 2019 Sep 20;7:204. doi: 10.3389/fcell.2019.00204 (PMC6771468; doi:10.3389/fcell.2019.00204)
Supplement: TABLE S1 — Primary antibodies for western blot. [file Table_1.DOCX]

**Table S1.** Primary antibodies for western blot

| Antibody  (Catalog number) | Company | dilution | Molecular Weight (kDa) |
| --- | --- | --- | --- |
| p-IκBα (ab92700) | Abcam, Cambridge, UK | 1:1000 | 36 |
| IκBα (ab32518) | Abcam | 1:5000 | 36 |
| p-p65 (ab76302) | Abcam | 1:1000 | 65 |
| P65 (ab32536) | Abcam | 1:10000 | 65 |
| p-p38(#4511) | Cell Signaling Technology, MA, USA | 1:1000 | 43 |
| p38 (#8203) | Cell Signaling Technology | 1:1000 | 40 |
| p-JNK(#4668) | Cell Signaling Technology | 1:1000 | 46, 54 |
| JNK (#8206) | Cell Signaling Technology | 1:1000 | 46, 54 |
| p-ERK(#4370) | Cell Signaling Technology | 1:1000 | 42, 44 |
| ERK (#4695) | Cell Signaling Technology | 1:1000 | 42, 44 |
| p-AKT (#4060) | Cell Signaling Technology | 1:1000 | 60 |
| AKT (#4685) | Cell Signaling Technology | 1:1000 | 60 |
