# Supplementary material for: Time-Course Transcriptome Analysis for Drug Repositioning in Fusobacterium nucleatum-Infected Human Gingival Fibroblasts
Source: Front Cell Dev Biol. 2019 Sep 20;7:204. doi: 10.3389/fcell.2019.00204 (PMC6771468; doi:10.3389/fcell.2019.00204)
Supplement: TABLE S2 — Primers sequences for quantitative real-time PCR (qRT-PCR). [file Table_2.DOCX]

**Table S2.** Primers sequences for quantitative real-time PCR (qRT-PCR)

| Gene | Primer sequences | | |
| --- | --- | --- | --- |
|  | 5ˊ~3ˊForward | 5ˊ~3ˊReverse | |
| GAPDH  IL-6  IL-8  CCL2  SOD2  PTGS2 | GCACCGTCAAGGCTGAGAAC  ATAACCACCCCTGACCCAAC  TCAGAGACAGCAGAGCACAC  CAGCCAGATGCAATCAATGCC  GGGATTGATGTGTGGGAGCA  GCTGTTCCCACCCATGTCAA | | TGGTGAAGACGCCAGTGGA  CCCATGCTACATTTGCCGAA  GGCAAAACTGCACCTTCACA  TGGAATCCTGAACCCACTTCT  CATAAAGAGCTTAACATACTCAGCA  AAATTCCGGTGTTGAGCAGT |

GAPDH: glyceraldehyde-3-phosphate dehydrogenase; IL: interleukin; SOD2: superoxide dismutase 2; PTGS2: prostaglandin-endoperoxide synthase 2
